# Therapiealternativen bei amyopathischer MDA5-positiver Dermatomyositis

**DOI:** 10.1007/s00108-023-01517-x

**Published:** 2023-05-30

**Authors:** Falk Schumacher, Maximilian Wollsching-Strobel, Doreen Kroppen, Sarah Bettina Schwarz, Johannes Strunk, Wolfram Windisch, Melanie Berger

**Affiliations:** 1 Klinik für Rheumatologie, Krankenhaus Porz am Rhein gGmbH, Urbacher Weg 19, 51149 Köln, Deutschland; 2 Lungenklinik Merheim, Kliniken der Stadt Köln GmbH, Köln, Deutschland; 3 Department Humanmedizin, Universität Witten/Herdecke, Witten, Deutschland

**Keywords:** Interdisziplinär, Idiopathische inflammatorische Myopathie, Interstitielle Lungenveränderungen, Cyclophosphamid, Mycophenolat-Mofetil, Interdisciplinary, Idiopathic inflammatory myopathy, Interstitial lung disease, Cyclophosphamide, Mycophenolate mofetil

## Abstract

Wir berichten über den Fall eines atypischen Therapieverlaufs bei amyopathischer MDA5-Antikörper-positiver Dermatomyositis mit Lungenbeteiligung. Aufgrund der schlechten Prognose erfolgte initial neben der Gabe von Prednisolon die frühzeitige Therapie mit Cyclophosphamid, gefolgt von Rituximab. Aufgrund des Therapieversagens erfolgte die Umstellung der Basistherapie auf Mycophenolat-Mofetil. Hierunter zeigte sich ein überraschend rascher positiver Verlauf bezüglich der Lungenveränderungen, der Hautveränderungen und der allgemeinen Krankheitsaktivität.

## Abstract (en)

We report a case of an atypical course of therapy in amyopathic MDA5-antibody-positive dermatomyositis with interstitial lung disease. Due to the poor prognosis, early therapy with cyclophosphamide followed by rituximab was carried out initially in addition to the administration of prednisolone. Due to therapy failure, treatment was switched to mycophenolate mofetil. This showed a surprisingly rapid positive course in terms of interstitial lung disease, skin manifestation, and general disease activity.

## Anamnese

Im November 2020 erfolgte die erstmalige Vorstellung einer 50-jährigen Patientin. Vorbekannt war ein effektiv therapierter Morbus Basedow und eine rezidivierende AV-Knoten-Reentry-Tachykardie (AVNRT) mit klinisch erfolgreicher Ablation. Die Patientin gab an, nie geraucht zu haben.

## Befund

**Figure 1:**
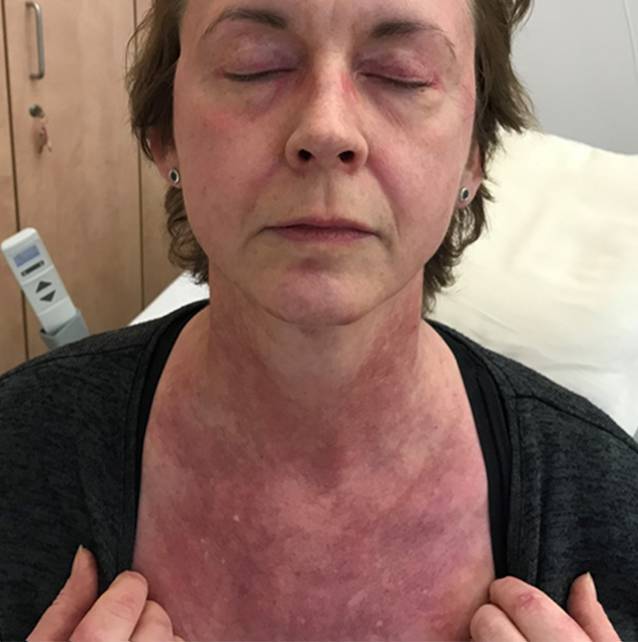

Seit 8 Wochen bestanden rasch progrediente Hautveränderungen im Sinne eines Erythems im Bereich des Dekolletés mit Juckreiz, rötlich-lividen Hautveränderungen im Bereich der Streck-/und Beugeseiten mehrerer Finger sowie papulösen Veränderungen der Handinnenflächen (Abb. 1, 2 und 3). Klinisch zeigten sich orale Aphthen, Arthralgien der Handgelenke beidseits, rezidivierendes Fieber bis 39 °C und ein ungewollter Gewichtsverlust von 5 kg in 6 Wochen sowie eine neu aufgetretene Belastungsdyspnoe.

**Figure 2:**
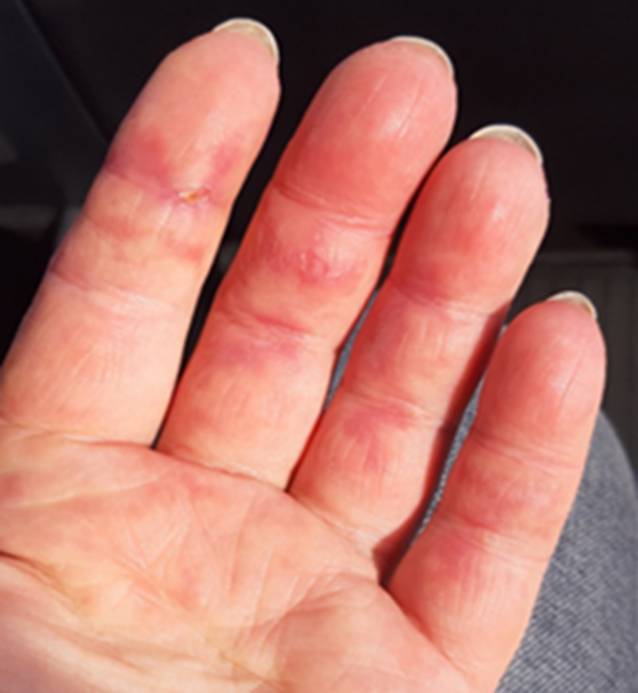

**Figure 3:**
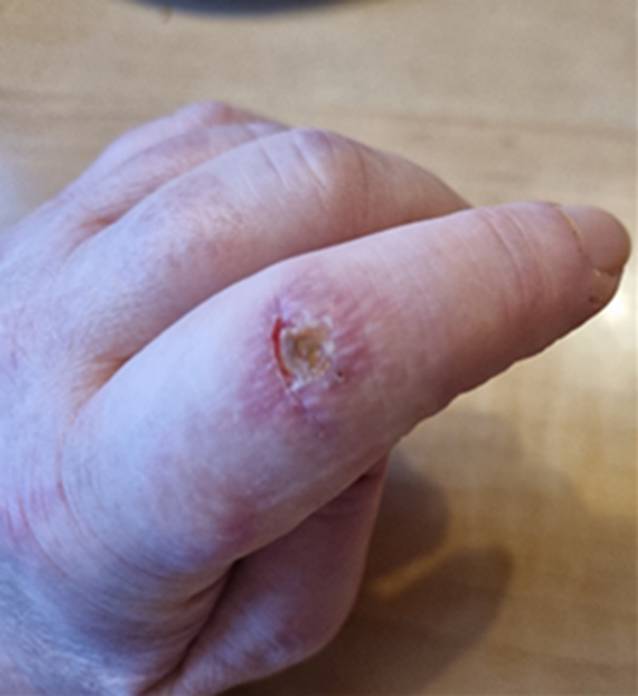

## Diagnose

**Figure 4:**
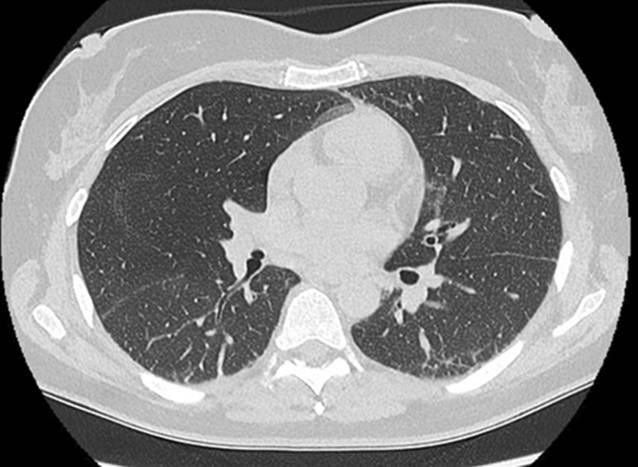

Laborchemisch bestanden keine relevant erhöhten Entzündungszeichen und normale Werte der Kreatinkinasen (CK). Es erfolgte eine umfangreiche Diagnostik ohne Hinweis auf eine infektiöse oder maligne Genese der Symptomatik. Initial zeigten sich in der Computertomographie (CT) des Thorax beginnende subpleural, peribronchial und basal betonte Konsolidierungen (Abb. 4). Lungenfunktionell zeigten sich eine restriktive Ventilationsstörung und eine Diffusionsstörung (Tab. 1). In der erweiterten Labordiagnostik ergaben sich der Nachweis von antinukleären Antikörpern (ANA) mit einem Titer von 1:100, positive Anti-Ro-52-Antikörper sowie gegen das Melanom-Differenzierungsantigen 5 gerichtete Antikörper (Anti-MDA5-Antikörper). Hinweise auf ein Sjögren-Syndrom wie eine Sicca-Symptomatik oder ein positiver Schirmer-Test bestanden zu diesem Zeitpunkt nicht. Wir stellten die Diagnose einer amyopathischen MDA5-positiven Dermatomyositis.

**Table 1:** 

	FVCex	TLC	FEV1/FVC	FEV1	TGV	RV	TLco	Kco
11/2020 (A)	2,5 l (66 %)	4,5 l (83 %)	77 % (89 %)	–	–	1,8 l (96 %)	–	–
01/2022 (B)	2,5 l (66 %)	4,1 l (76 %)	75 % (95 %)	1,9 l (64 %)	2,2 l (79 %)	1,6 l (85 %)	47 %	76 %
03/2022 (C)	2,7l (72 %)	4,1l (76 %)	77 % (97 %)	2,1 l (70 %)	2,3 l (81 %)	1,4 l (74 %)	50 %	73 %
06/2022 (D)	2,8 l (74 %)	5,0 l (91 %)	77 % (97 %)	2,2 l (74 %)	3,2 l (111 %)	2,1 l (110 %)	56 %	77 %

*FVCex* forcierte exspiratorische Vitalkapazität, *TLC* totale Lungenkapazität, *FEV1* Einsekundenkapazität, *TGV* thorakales Gasvolumen, *RV* Residualvolumen, *TLco* Kohlenmonoxid-Transferfaktor, Diffusionskapazität *Kco* Transferkoeffizient

## Therapie und Verlauf

**Figure 5:**
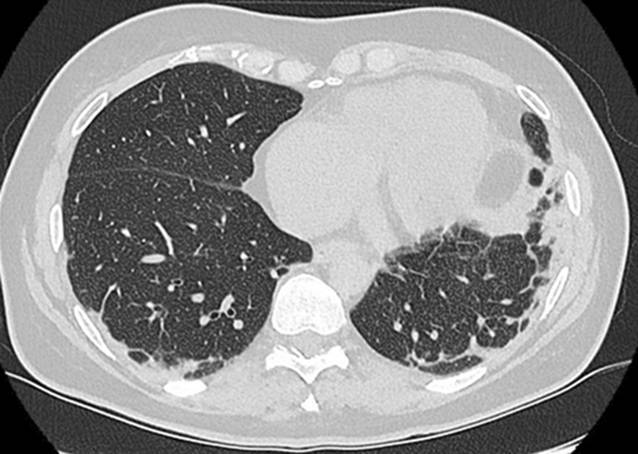

Es erfolgte eine Therapie mit Prednisolon (1 mg/kgKG) und Azathioprin (150 mg/Tag) über 4 Monate. Unter der Predisolon-Reduktion bis 20 mg/Tag zeigten sich rezidivierende Gottron-Papeln, ein erneutes heliotropes Exanthem sowie eine beginnende Muskelschwäche der proximalen Extremitäten. Bei klinisch deutlich progredienter Belastungsdyspnoe und auskultatorischem Knisterrasseln zeigten sich im CT-Thorax ebenfalls progrediente Konsolidierungen, sodass eine Therapie mit Cyclophosphamid (dreimalige Gabe von 1000 mg) erfolgte (Abb. 5). Die geplante Gabe von 6 Infusionen à 1000 mg Cyclophosphamid wurde aufgrund der rasch progredienten Verschlechterung der respiratorischen und kutanen Manifestationen somit zugunsten eines Wechsels der Therapiestrategie unterbrochen. Bei einem Therapieversagen von Cyclophosphamid und weiter progredienter Lungenbeteiligung in der CT nach 4 Monaten erfolgte eine zweimalige Gabe von 1000 mg Rituximab (im Abstand von 2 Wochen). Nach einem weiteren Intervall von 4 Monaten war keine Prednisolon-Reduktion unter eine Tagesdosis von 30 mg möglich. Es erfolgte bei weiter progredienter Belastungsdyspnoe eine Umstellung der Basistherapie auf Mycophenolat-Mofetil (MMF), 2 g/Tag.

**Figure 6:**
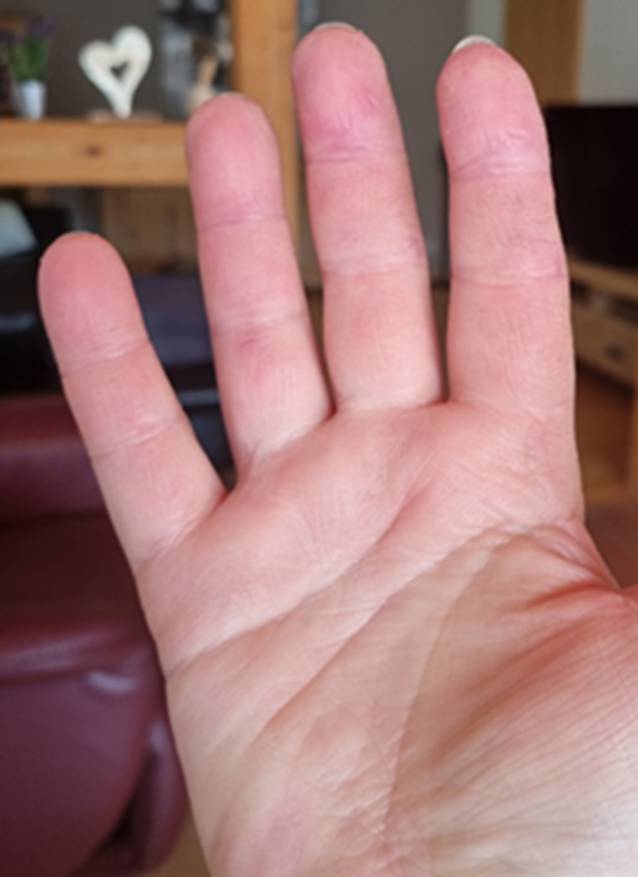

Erstmalig war es nach 3 Monaten unter der Therapie mit MMF zu einer klinischen Besserung gekommen. Sowohl die Hautveränderungen als auch die Arthralgien/Myalgien und die Belastungsdyspnoe zeigten sich deutlich gebessert (Abb. 6 und 7). Zu Fieberschüben ist es nicht mehr gekommen. Nach 5 Monaten war eine Dosisreduktion der Prednisolon-Therapie auf 7,5 mg/Tag möglich.

**Figure 7:**
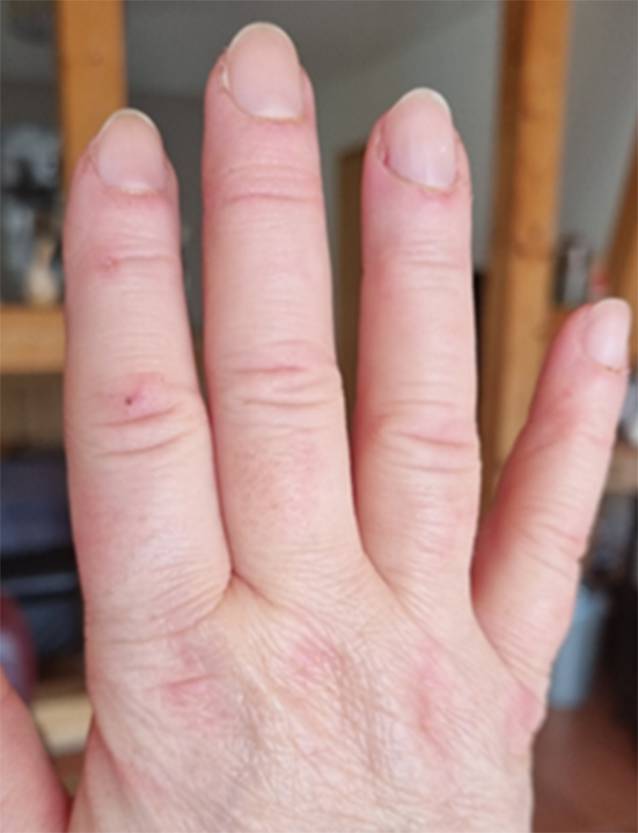

**Figure 8:**
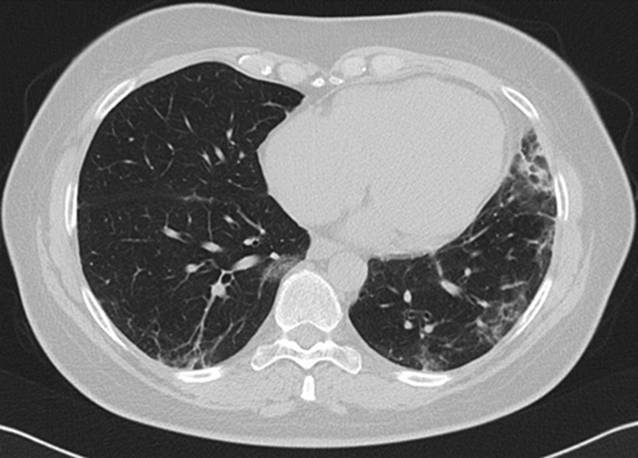

**Figure 9:**
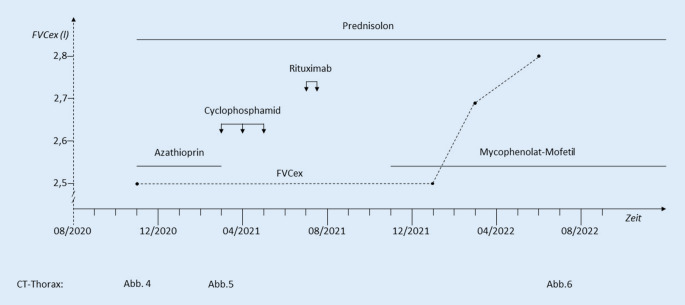

In der Computertomographie (CT) des Thorax zeigte sich nach 8 Monaten Therapie mit MMF eine Größenregredienz der subpleural, peribronchial und basal betonten Konsolidierungen (Abb. 8). Lungenfunktionell zeigte sich im Verlauf von 6 Monaten eine Besserung der restriktiven Ventilationsstörung sowie der Diffusionsstörung (Tab. 1). Somit konnte ein positiver Verlauf unter MMF bei therapierefraktärer MDA5-assoziierter Dermatomyositis mit rasch progredienter Lungenbeteiligung gezeigt werden (Abb. 9).

## Diskussion

Die interstitiellen Lungenveränderungen bei amyopathischer MDA5-Antikörper-positiver Dermatomyositis bestimmen maßgeblich die Morbidität und die Mortalität dieser Patientengruppe [1]. Aufgrund der Seltenheit der Erkrankung ist die Evidenz zur Etablierung von Handlungsempfehlungen begrenzt. Die in der Literatur beschriebene 6‑Monats-Überlebensrate liegt bei 40 % [2]. Aufgrund der hohen Mortalität wird häufig auch eine aggressive Kombination von immunmodulierenden Therapien eingesetzt [1]. Die Therapie mit MMF in Kombination mit Prednisolon tritt somit bei den Therapieoptionen häufig in den Hintergrund. Einige Fallberichte beschreiben eine Verbesserung der interstitiellen Lungenveränderungen unter der Therapie mit Rituximab nach Versagen anderer immunsuppressiver Therapien wie Cyclophosphamid [3, 4]. Dieser Verlauf ließ sich in unserem Fall nicht bestätigen. Der positive Effekt der Therapie mit MMF konnte allerdings in anderen Fallberichten auch in der Erstlinientherapie bestätigt werden [5].

In der Literatur konnte ein positiver Nutzen eines kombinierten Therapieschemas aus hoch dosierten systemischen Glukokortikoiden und anderen Immunsuppressiva wie Calcineurin-Inhibitoren und/oder Cyclophosphamid gezeigt werden [6]. In dem von uns berichteten Fall wurde zum Zeitpunkt der Therapie mit Cyclophosphamid von einer Ergänzung der Therapie um einen Calcineurin-Inhibitor aufgrund der möglichen unerwünschten Nebenwirkung einer zeitgleichen Medikation und der bestehenden Therapiealternativen vorerst abgesehen.

In unserem Fall erfolgte eine sequenzielle Therapie. Dennoch wäre auch ein verzögertes Therapieansprechen auf Rituximab und somit eventuell auch eine synergistische Wirkung von Rituximab und MMF möglich. [7].

Auch wenn die Literatur weitere vielversprechende Therapieoption mit neuen Substanzen wie JAK-Inhibitoren (z. B. Tofacitinib) bietet [8], sollte MMF in die Entscheidung des individualisierten Therapieregimes von Patienten mit MDA5-positiver Dermatomyositis mit Lungenbeteiligung eingeschlossen werden.

## Fazit für die Praxis

Mycophenolat-Mofetil stellt eine relevante Therapiealternative bei therapierefraktärer amyopathischer MDA5-positiver Dermatomyositis mit Lungenbeteiligung dar.
